# Sweat-Sensing Patches with Integrated Hydrogel Interface for Resting Sweat Collection and Multi-Information Detection

**DOI:** 10.3390/bios15060342

**Published:** 2025-05-29

**Authors:** Lei Lu, Qiang Sun, Zihao Lin, Wenjie Xu, Xiangnan Li, Tian Wang, Yiming Lu, Huaping Wu, Lin Cheng, Aiping Liu

**Affiliations:** 1 Zhejiang Key Laboratory of Quantum State Control and Optical Field Manipulation, Department of Physics, Zhejiang Sci-Tech University, Hangzhou 310018, China; lulei0411@126.com (L.L.); dz1447368777@163.com (Q.S.); greatsterling123@gmail.com (Z.L.); xwjloveb@outlook.com (W.X.); 13674232220@163.com (X.L.); 2023326690008@mails.zstu.edu.cn (Y.L.); 2 Key Laboratory of Special Purpose Equipment and Advanced Processing Technology, Ministry of Education and Zhejiang Province, College of Mechanical Engineering, Zhejiang University of Technology, Hangzhou 310023, China; wuhuaping@gmail.com

**Keywords:** sweat sensor, hydrogel interface, microfluidic platform, resting sweat rate, multi-information detection

## Abstract

Sweat analysis represents an emerging non-invasive approach for health monitoring, yet its practical application is hindered by challenges such as insufficient natural sweat secretion and inefficient collection. To overcome these limitations, this study develops a hydrogel sheet composed of agarose and glycerol, which efficiently facilitates resting sweat collection without external stimulation when integrated into the microfluidic channels of a sweat-sensing patch. The microfluidic sweat-sensing patch, fabricated with laser-cut technology, features a sandwich structure that enables the measurement of sweat rate and chloride ion concentration while minimizing interference from electrochemical reactions. Additionally, a colorimetric module utilizing glucose oxidase and peroxidase is also integrated into the platform for cost-effective and efficient glucose detection through a color change that can be quantified via RGB analysis. The hydrogel interface, characterized by its optimal thickness and water content, exhibits superior absorption capability for efficient sweat collection and retention, with a negligible effect on the dilution of sweat components. This hydrogel-interfaced microfluidic platform demonstrates high efficiency in sweat collection and multi-biomarker analysis, offering a non-invasive, real-time solution for health monitoring. Its low-cost and wearable design highlights its potential for broad applications in personalized healthcare.

## 1. Introduction

The escalating demand for non-invasive, real-time health monitoring has driven substantial advancements in wearable biosensing technologies. Among various biofluids [1,2,3,4,5], sweat has emerged as an especially promising medium for health monitoring due to its accessibility, continuous secretion, and rich content of physiological biomarkers, such as electrolytes and metabolites [6,7,8,9]. Traditional methods for sweat collection, however, often rely on external stimuli, including iontophoresis [10,11,12], exercise-induced sweating [13], or thermally induced perspiration [14]. Although these approaches are effective, they are invasive, uncomfortable, and impractical for routine or continuous monitoring, or are unsuitable for individuals with mobility difficulties, thereby limiting their applicability in wearable health monitoring systems. In contrast, natural thermoregulated sweating does not require active stimulation [15,16] and can occur steadily during prolonged periods of sitting or daily activities. The lower sweating rate also minimizes dilution of secreted metabolites, allowing for a more accurate correlation between biochemicals in blood and sweat [17]. Consequently, the detection of natural sweat provides a more reliable reflection of the body’s physiological health information [18]. However, natural sweat secretion at rest is typically insufficient for effective sampling, posing a significant challenge for the development of sweat sensing platforms. This limitation underscores the need for innovative designs to enhance sweat collection without relying on external stimuli. When evaluating potential solutions, traditional passive collection methods often fail to efficiently manage low-volume fluids, leading to inconsistent sample delivery or evaporation losses. In contrast, microfluidic systems offer precise control over fluid dynamics at the microscale, enabling reliable manipulation of the scarce natural sweat output.

To accurately deliver collected sweat to sensing interfaces, microfluidic systems have become indispensable components of wearable sweat-sensing platforms [19,20,21]. These systems efficiently collect minute quantities of sweat and direct it in a controlled manner to well-defined sensing regions, thereby minimizing sample loss and contamination. Microfluidic channels, often constructed using adhesive layers embedded with conductive materials, provide a compact and efficient means of integrating multiple sensing modalities [22]. For example, electrochemical sensors embedded within these microfluidic channels enable the detection of biomarkers while preserving sample integrity [23,24]. However, careful design is essential to minimize interference and ensure optimal interaction between the sweat sample and the sensing element.

Sweat composition is highly complex, with analyte concentrations influenced by individual physiology, environmental conditions, and sweat secretion rates [25,26]. These variables pose significant challenges for the accurate detection and quantification of biomarkers. Electrochemical sensors, which convert biochemical signals into electrical outputs [27,28], have emerged as a promising solution for the quantitative detection of biomarkers, especially electrolytes in sweat [29,30]. For example, Nyein et al [31] developed a microfluidic sensing patch via a roll-to-roll process that measured real-time sweat parameters such as [Na^+^], [K^+^], [glucose], and sweat rate during exercise and chemically induced sweating. In addition to electrochemical sensing, colorimetric methods provide a complementary approach for sweat analysis, particularly in the detection of glucose levels [32,33]. Colorimetric sensors leverage visible color changes resulting from the reaction between analytes and chromogenic reagents, allowing for simple and cost-effective detection without the need for an external power source. For instance, Xiao et al. [34] developed a wearable colorimetric sensor based on a microfluidic chip that could perform five parallel measurements simultaneously, presenting a linear range of 0.1–0.5 mM for sweat glucose with a detection limit of 0.03 mM, allowing it to sensitively detect small differences in sweat glucose concentration between fasting and post-glucose intake states. Similarly, Cheng et al. [35] developed a paper-based microfluidic electrochemical colorimetric sensor, which demonstrated high accuracy and reliability for human sweat analysis. These studies underscore the potential of colorimetry for non-invasive diabetes prevention and management. While unimodal sensing approaches (e.g., electrochemical-only [36] or colorimetric-only [37]) simplify device architecture, they inherently struggle to decouple confounding factors such as sweat rate fluctuations and interferent cross-talk. To overcome this, dual-modal sensing—combining electrochemical and colorimetric principles—provides a synergistic solution, namely electrochemical sensors enable dynamic tracking of high-frequency parameters (e.g., electrolytes), while colorimetric methods offer stable quantification of low-concentration metabolites (e.g., glucose) through spatially resolved signal amplification. Given the complementary advantages of electrochemical sensing and colorimetry, integrating these technologies presents a promising strategy for developing a versatile sweat-sensing platform.

**Figure 1:**
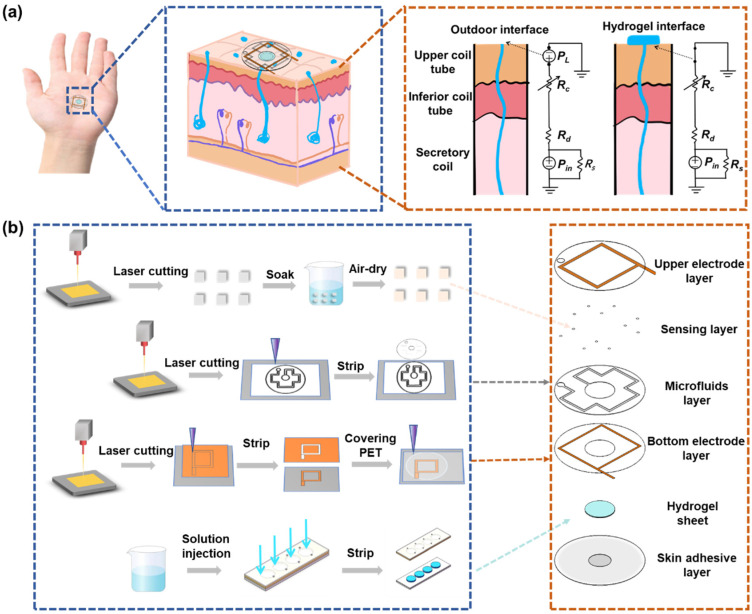
Schematic diagram of microfluidic sweat sensing patch. (**a**) Model for resting sweat detection and theoretical analysis of natural sweat collection via hydrogel interface. (**b**) Flowchart illustrating the fabrication process along with exploded view of the sweat sensing patch.

In this study, we propose a hydrogel-interfaced microfluidic platform specifically designed to overcome the limitations associated with natural sweat collection and multi-modal sensing (Figure 1a). A hydrogel sheet, composed of agarose and glycerol, enables rapid and efficient sweat absorption without requiring external stimuli, channeling the collected sweat into microfluidic channels for subsequent analysis. This platform incorporates electrochemical sensors for quantifying sweat rate and chloride ion concentration, as well as a colorimetric module for glucose detection via enzymatic reactions and RGB-based quantification (Figure 1b). The developed hydrogel-interfaced microfluidic patch serves as a versatile and reliable non-invasive tool for continuous health monitoring, offering broad applications in clinical diagnostics, fitness tracking, and personalized medicine.

## 2. Material and Methods

### 2.1. Reagents and Materials

Dust-free paper (WIP-0609) was purchased from Deleo Co., Ltd. (Chadderton, UK). Double-sided tape was received from 3M China Co., Ltd. (Shanghai, China), with a thickness of 0.15 mm per layer. Polyethylene terephthalate (PET) film was provided from Dongguan Fangcheng Plastic Co., Ltd. (Dongguan, China). Low-adhesion tape and copper tape were obtained from Shenzhen Wangxing Tape Co., Ltd. (Shenzhen, China). Agarose, glycerol, PBS, glucose oxidase (GOx), and ethanol (C_2_H_5_OH) were purchased from Aladdin Chemical Reagents (Shanghai, China). The glucose assay kit was provided by Leagene (Hong Kong, China).

### 2.2. Fabrication of Hydrogel Sheet

A mold was assembled with a glass sheet as the substrate, double-sided adhesive tape as the middle layer, and PET film as the sealing layer. The double-sided tape layer and PET layer were precisely cut with a UV laser cutter to form a microfluidic chamber with an 8 mm diameter and an inlet/outlet ports with a 1 mm diameter, respectively. The thickness of the hydrogel could be controlled by varying the number of double-sided tape layers. Then a solution of 2% agarose and 50% glycerol in deionized water was prepared and heated in a water bath at 80 °C for 3 h until complete dissolution. The solution was immediately injected into the preheated mold (maintained at 80 °C on an electric hot plate). After sufficient cooling and curing, the hydrogel sheet was obtained by carefully peeling off the double-sided adhesive tape and PET layer from the glass substrate. The prepared hydrogel sheets were stored in phosphate buffer solution at 4 °C for subsequent use.

### 2.3. Preparation of Colorimetric Sensing Layer

A colorimetric method was used to detect glucose concentration in sweat using enzyme-based reagents [38]. First, the phenol reagent and enzyme reagent were mixed at a 1:1 ratio, followed by the addition of an equal volume of glucose oxidase (GOD) to create a fully homogenized GOD-POD working solution. Squares with a side length of 0.5 mm were fabricated from dust-free paper using ultraviolet laser cutting. The prepared paper squares were subsequently immersed in the GOD-POD working solution at 4 °C for 3 h to allow thorough soaking. After immersion, the squares were carefully removed, dried, and stored in a light-proof environment at 4 °C to preserve their reactivity and stability. These functionalized colorimetric squares were then integrated into the microfluidic channels for glucose detection.

### 2.4. Preparation of Microfluidic Sweat Sensing Patch

Draw the overall structural design of the sensor in AutoCAD 2024 software, which included the upper electrode layer, colorimetric sensing layer, microchannel layer, lower electrode layer, hydrogel layer, and adhesive layer from top to bottom (Figure 1b). For the copper electrode layer, a 3 cm × 3 cm copper tape was first cut and adhered to the PET substrate. The laser parameters were then adjusted to cut out a 1 mm wide copper tape, after which the excess material was removed. Then, a lower electrode layer with a width of 600 μm was obtained using the same method. To secure the upper electrode layer, tape was used to fix it in place, followed by cutting a 3 cm × 3 cm piece of 3M tape to cover the upper electrode layer. After sticking, the release paper of the 3M tape was removed, and a laser was used to cut a microfluidic channel through the 3M tape, with a channel width of 0.6 mm, a central collection pool of 8 mm, and an outlet of 2 mm. Finally, the excess 3M tape was peeled off. For sensor assembly, the colorimetric sensor patch was carefully embedded into the microfluidic channel using tweezers to reach the predetermined position. Then, the lower electrode layer was pasted to the appropriate position on the underside of the microfluidic channel layer. Subsequently, the prepared hydrogel sheet was placed in the collection groove of the microfluidic channel layer, and finally the adhesive layer was pasted under the lower electrode to form a sweat sensor. The details about the dimensions of the sensor are shown in Figure S1.

### 2.5. Characteristic

The morphological characteristics of the hydrogel sheets were observed using a scanning electron microscope (SEM-4800 Carl Zeiss SMT Pte. Ltd., Guangzhou, China). A precision electronic balance (AUW220, Shanghai Fangrui Instrument Factory, Shanghai, China) was used to test and evaluate the sweat absorption performance of the hydrogel sheets. The AD5933 electrochemical detection system was constructed based on the principle of impedance spectroscopy. To test the patch sensing performance in vitro, NaCl solutions at different concentrations and rates were injected using a syringe pump (LSP01-3A, Baoding Langer Constant Flow Pump Co., Ltd., Baoding, China). The glucose concentration in sweat was measured using the glucose oxidase (GOD) coupled assay. After the color change of the patch was completed, the SONY camera (FDRAX60 SONY Camera Co., Ltd., Hongkong, China) was used to take pictures and record images. The RGB value of the maximum color block was obtained by the Color Picker software (version 1.6, Banlige Software Co., Ltd., Xi’an, China) for analysis. For in situ experiments on sweat rate, sweat chloride concentration, and glucose, sweat-sensing patches were attached to different parts of two volunteers exposed to 25 °C at room temperature. During the on-body tests of the sweat glucose concentration for each volunteer, we also measured blood glucose with a commercial blood glucose meter (Yuwell 590, Shanghai, China).

## 3. Result and Discussion

### 3.1. Collection Principle of Natural Sweat via Hydrogel Interface

Under natural conditions, sweat secretion occurs at a relatively low rate. However, studies have demonstrated that the densities of activated sweat glands in the fingers, palms, and backs of hands are approximately 532, 305, and 167 glands·cm^−2^, respectively, with corresponding natural sweat rates of 0.15, 0.14, and 0.07 mg·cm^−2^·min^−1^ [39]. In contrast, the sweat evaporation rate ranges from 0.0197 to 0.0255 mg·cm^−2^·min^−1^ [40]. These observations highlight that the natural sweat rate consistently exceeds the evaporation rate, providing a theoretical basis for the collection and analysis of natural sweat. Figure 1a illustrates the first-order microfluidic model of the sweat gland. In this model, *P_in_* represents the hydrostatic osmotic pressure generated by the accumulation of Na^⁺^ and Cl^⁻^ ions in the secretory cavity, which drives sweat from the gland to the skin surface, forming sweat droplets [41]. The fluid resistances of the upper coil, dermis, and secretory coil are denoted by *R_c_*, *R_d_*, and *R_s_*, respectively. Sweat secretion is opposed by the Laplace pressure (*P_L_*) caused by surface tension, arising from sweat droplets with a curvature radius *R*. To sustain natural sweating, *P_in_* must exceed *P_L_*. As sweat secretion proceeds, *P_in_* decreases due to ion loss, necessitating it to surpass the *P_L_* threshold to trigger the next secretion cycle, thereby enabling continuous sweating. To address the obstruction posed by *P_L_*, a hydrogel sampling interface is proposed at the skin pores. This interface establishes a continuous fluid path linking the secretory coil to the sampling reservoir and reduces *P_L_* to nearly zero [42]. Comparative experiments of sensor sweat collection with and without hydrogels are shown in Figure S2. Consequently, sweat secretion can occur even at lower *P_in_* levels. Therefore, the hydrogel interface can rapidly sample natural sweat without external stimulation and guide it into the microfluidic channel for analysis, effectively overcoming the challenge of low sweat secretion rates under natural conditions.

### 3.2. Properties of Hydrogel Sheets

**Figure 2:**
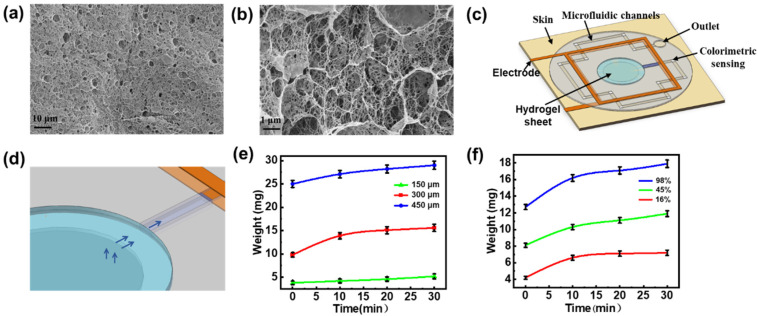
Preparation and characterization of hydrogel sheet. (**a**,**b**) SEM images of hydrogel sheet. (**c**) Schematic representation of the sweat sensing patches. (**d**) Schematic representation of sweat collection. (**e**) Sweat absorption performance of hydrogel sheets (90% water content) with varying thicknesses of 150 μm, 300 μm, and 450 μm. (**f**) Sweat absorption properties of hydrogel sheets (300 μm thickness) with different water contents.

Owing to its distinctive structural characteristics, the hydrogel is utilized as a sweat collection layer in the sensing patch, facilitating the diffusion of biochemical components from sweat for subsequent analysis. SEM characterization (Figure 2a,b) reveals that the hydrogel’s three-dimensional (3D) network structure creates numerous pores, thereby increasing its specific surface area and enhancing the adsorption efficiency. The porous polymer network, combined with increased surface roughness, provides additional adsorption sites, thus improving the hydrogel’s overall adsorption performance [43]. The sweat collection concept using the sweat-sensing patch is shown in Figure 2c,d. The collection cell in the patch’s inlet contains a hydrogel sheet doped with a solute (glycerol) at a higher concentration than that found in body sweat, thereby generating an osmotic driving force that facilitates the entry of sweat into the microfluidic channel. Subsequently, the microfluidic channel captures this osmotically driven sweat and continuously delivers it to the sensor. Considering that the adsorption properties of hydrogels can be influenced by sample thickness, we conducted sweat adsorption experiments using hydrogel sheets with various thicknesses (150 μm, 300 μm, and 450 μm; see Figure S3). These sheets were applied to skin covered with an elastic waterproof membrane, and their weight changes were recorded at 10 min intervals. The experimental results (Figure 2e) indicate that the 150 μm-thick hydrogel sheet exhibits minimal weight change, suggesting inadequate perspiration absorption. In contrast, the 300 μm-thick sheet presents the highest absorption efficiency, with the most significant weight increase during the first 10 min. Following this initial phase, the rate of change stabilizes, suggesting that adsorption and desorption has reached equilibrium. The 450 μm-thick sheet also displays enhanced absorption capacity but requires more time to reach equilibrium. Based on these findings and sensor assembly requirements, the 300 μm-thick hydrogel sheet is selected as the optimal interface for natural sweat absorption.

In addition, the water content of hydrogels establishes a dynamic equilibrium between the adsorption and desorption processes, which can influence both adsorption time and sweat transfer efficiency [44]. We evaluated the impact of water content on the sweat-absorbing properties of 300 µm-thick hydrogel sheets. Water content was adjusted to 98%, 45%, and 16% by either natural evaporation or baking at 60 °C for 30 to 60 min. The results (Figure 2f) demonstrate that the hydrogel with 98% water content exhibits superior swelling properties, absorbing the most sweat within the first 10 min, and maintaining a high absorption rate over 30 min, thereby demonstrating optimal performance for sweat collection and retention. In contrast, the patch with 45% water content shows reduced swelling capacity and lower sweat absorption efficiency. The sheet with 16% water content, while exhibiting better stability and mechanical properties [45], displays minimal weight change after 10 min, indicating poor sweat extraction capability and thus the weakest absorption properties. Despite potential dilution effects from higher water content, the 300 μm-thick hydrogel sheet with 98% water content is still preferred as the collection layer for natural resting sweat, ensuring both effective absorption and subsequent analysis. At the same time, we tested the mechanical stability and absorption efficiency stability of the patch [46]. As shown in Figure S4, the weight retention was greater than 95% for 120 h at room temperature (25 °C), the hydrogel morphology was intact, there was no cracking on the surface, and the device as a whole showed good mechanical stability. The sweat rate test of the sensors was performed under the same test conditions after 1, 3, and 5 days of placement, and it can be seen that the repeatability of the sweat rate test is good. The results indicate that the patch maintains a stable absorption efficiency and mechanical stability under prolonged conditions.

### 3.3. In Vitro Collection and Analysis of Sweat Using Microfluidic Sweat Sensing Patches

For the microfluidic sweat sensing patch, the microfluidic channel layer was created by cutting double-sided adhesive tape with a UV laser. Laser ablation, which affects the roughness of the inner channel walls, can influence sweat flow within the channel. To optimize the laser cutting process, we systematically varied the scanning rate to 10 mm·s^−1^, 30 mm·s^−1^, 40 mm·s^−1^, and 50 mm·s^−1^, and examined the resulting roughness of the channel walls. As shown in Figure S5, increasing the scanning rate consistently reduces the roughness of the channel walls. At a scanning rate of 50 mm·s^−1^, the channel surface becomes smoother, likely due to the shorter dwell time of the laser at any given point, thereby minimizing the extent of ablation. Consequently, this scanning rate is selected for cutting the double-sided adhesive tape. Next, we investigated the effects of channel width on the ablation process. Microfluidic channels with widths ranging from 0.2 mm to 1 mm were processed while keeping the laser parameters constant (wavelength: 355 nm, pulse width: 1 μs, scanning rate: 50 mm s^−1^). Optical microscope images of the processed channels, as shown in Figure S6, reveals that narrower channels (e.g., 0.2 mm) exhibit significant ablation marks and adhesion issues due to overlapping laser spots. In contrast, channels with widths of 0.6 mm or greater have smoother inner walls with minimal ablation, suggesting that such channel widths do not significantly impact sweat flow.

For optimal microfluidic channel design, we also considered flow resistance. Previous studies [47,48] have shown that the flow resistance of a channel is determined by the following equation

(1) $$R_{c}=\frac{8\mu_{S}L_{S}}{wh}{\left(\frac{1}{w}+\frac{1}{h}\right)}^{2}.$$

Here, *w* is the channel width, *h* is the channel height, *L*_s_ is the length of the sweat-filled microfluidic channel, *μ_s_* is the viscosity of sweat (0.7225 × 10⁻^3^ Pa·s at 35 °C) [49]. According to this formula, the flow resistance increases with the length of the sweat-filled section, while it decreases as the channel width or height increases. Since microfluidic devices typically operate at the micrometer scale, we consider channel dimensions ranging from 0.05 mm and 1 mm. We calculated the flow resistance for a 0.6 mm wide, 100 mm long, channel with various height. As shown in Figure S7a, flow resistance decreases rapidly when the channel height is higher than 0.2 mm, with minimal change observed beyond this point. Similarly, we examined the effect of channel width on flow resistance by varying the width while keeping the height at 0.3 mm. As shown in Figure S7b, the flow resistance decreases significantly when the channel width is higher than 0.3 mm, with slower changes for wider channels. Given that natural sweat secretion rates are lower compared to exercise-induced sweat, we aim to minimize the channel volume to enhance absorption efficiency. Therefore, a channel width of 0.6 mm was chosen, balancing smoothness and sweat absorption efficiency. Finally, we assessed the effect of channel length on flow resistance, as shown in Figure S7c. With increasing channel length from 10 mm to 320 mm, the flow resistance increases by only 1 kPa·s·mm⁻^3^, indicating a negligible impact on resistance at typical lengths used in our experiments. Based on these findings, we determine the optimal microfluidic channel dimensions to be 600 μm in width and 300 μm in height, with the length adjusted according to specific experimental needs.

**Figure 3:**
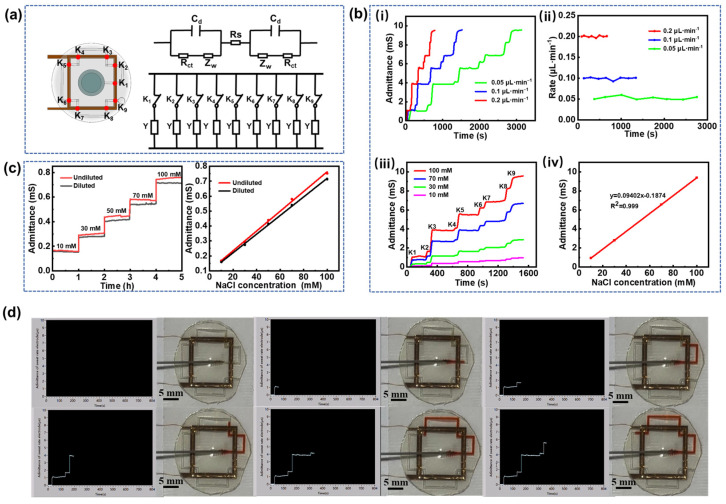
In vitro sweat analysis. (**a**) Equivalent circuit model of sweat at adjacent trigger points. (**b**) (i) Electrode conductance curves at different injection rates, (ii) sweat flow rates at different injection rates, (iii) electrode conductance curves at varying NaCl concentrations, and (iv) fitting curves correlating electrode conductance to solution concentration. (**c**) Stepwise electrode conductance curves with and without hydrogel at different Cl^−^ concentrations along with corresponding calibration curves for Cl^−^ concentration versus conductance. (**d**) Interface for sweat rate measurement and its corresponding physical diagram.

When sweat flows into the microfluidic channel from the collection holes and reaches the trigger point locations (indicated by red dots in Figure 3a), it simultaneously infiltrates both the upper and lower copper electrodes. This infiltration forms a conductive pathway between the electrodes due to the high concentration of electrolytes in the sweat. The equivalent circuit for each trigger point, shown in Figure 3a, comprises several components: *C_d_* denotes the double-layer capacitance at the electrode-electrolyte interface at the trigger point, *R_ct_* denotes the charge transfer impedance, *Z_w_* represents the diffusion impedance, and *R_s_* is the solution resistance. At each trigger point, the circuit can be simplified to an equivalent conductance *Y*. When sweat flows through a trigger point, it introduces a conductance in parallel with the existing circuit, leading to a sharp increase in the overall conductance of the upper and lower electrodes. However, when the sweat flows between two adjacent trigger points, the conductance remains constant. Consequently, the conductance curve exhibits a step-like pattern. By analyzing the duration of each step, the time required for the sweat to reach each trigger point can be determined, allowing for the calculation of the average sweat rate during the time interval

(2) $$Q_{i}=\frac{V_{i}}{t_{i}-t_{i-1}}.$$

Here, *V_i_* is the volume of sweat in the *i*-th channel(*V_i_* = w·h·*i*), *t_i_* represents the time of the *i*-th trigger point, and the average sweating rate can be recorded as *Q_i_* using the aforementioned equation.

The primary electrolytes in sweat include sodium ion (Na^+^) and chloride ions (Cl^−^), along with lesser concentrations of K^+^, NH^4+^, and Ca^2+^. Notably, the predominant anions in sweat are Cl^−^ and bicarbonate (HCO^3−^), but the concentration of Cl^−^ is approximately 20 times higher than that of HCO^3−^ [50]. As shown in Supplementary Figure S8, NaCl, KCl, and CaCl_2_ solutions with the same Cl^−^ concentration have the same admittance values, so it is clear that electrode admittance is independent of cation type and only dependent on Cl^−^ concentration. Given the balance between anions and cations, sweat conductivity is predominantly determined by Cl^−^ concentration. Since conductivity is the reciprocal of impedance, measuring the impedance between electrodes allows for the calculation of conductivity, thereby enabling the detection of Cl^−^ concentration. Based on this, we utilized the AD5933 impedance measurement chip to construct a microfluidic sensing platform. The chip integrates a frequency generator and a 12-bit analog-to-digital converter (ADC). The output port transmits a sinusoidal excitation signal with an adjustable amplitude, while the response signal is sampled by the ADC. The data are subsequently processed by a digital signal processor (DSP) to perform a discrete Fourier transform (DFT), as illustrated in Figure S9. The DFT algorithm yields both the real part (R) and imaginary part (I) of the impedance at each frequency. These values are computed and retrieved from the register via a serial I2C interface. To minimize the influence of DC signals on the excitation signal, a high-frequency filter capacitor is used to remove the DC component, thereby preventing electrode polarization. The measured impedance values are then transmitted to a host computer via a microcontroller and visualized using LabVIEW (version 2019) (Figure S10).

In vitro simulation experiments were conducted by injecting a 100 mM NaCl solution into the microfluidic channel from the sweat collection holes at injection rates of 0.05, 0.1, and 0.2 μL·min^−1^. The electrode conductance data obtained from the microfluidic sensing test platform reveal that the conductance values at each trigger point remain consistent regardless of the injection rate, indicating that electrode conductance is independent of the injection rate (Figure 3b(i)). Additionally, the conductance remains unchanged between adjacent trigger points, corroborating the theory of sweat rate measurement. By substituting the duration of each step and the volume of the solution into Equation (2), the sweat rate at each point was calculated (Figure 3b(ii)). The results confirm that the measured flow rate of NaCl solution in the microfluidic channel is consistent with the injection rate, validating the accuracy of the measurement technique. Furthermore, the NaCl solutions with concentrations of 10 mM, 30 mM, 70 mM, and 100 mM were sequentially injected into the microfluidic channel at a constant flow rate of 0.1 μL·min^−1^. As expected in Figure 3b(iii), the conductance remains stable between trigger points, and increases proportionally with the NaCl solution concentration at each trigger point, demonstrating a highly linear relationship with *R*^2^ = 0.999 (Figure 3b(iv)). Movie S1 in the Supporting Information demonstrates the microfluidic testing process at an injection rate of 0.1 μL·min^−1^. As the solution passes each trigger point, its conductivity increases abruptly, which is visualized in real time via the LabVIEW interface. Figure 3d provides a screenshot of the visualized interface along with a practical diagram of the in vitro injection process. The observed step-like pattern in the conductivity curve aligns with the expected measurement theory. It should be noted that sweat undergoes adsorption and desorption by the hydrogel sheet before entering the microfluidic channel, leading to some inevitable dilution of sweat components due to the hydrogel’s high water content. To quantify the dilution effect of the hydrogel, chloride ions were selected as the target analyte. A control experiment was conducted on the microfluidic sensing patches with and without the hydrogel interface to evaluate its impact on Cl^−^ concentration. As shown in Figure 3c, the electrode conductance remains stable over 1 h, indicating the sensor’s consistent performance. However, the conductance of the hydrogel-diluted solution is slightly reduced, suggesting a decrease in Cl^−^ concentration. The deviations of the linear fitting curve between Cl^−^ concentrations and conductance remain within 4% to 7%, indicating that the use of the 300 μm-thick hydrogel sheet as an interfacial layer has a negligible effect on the dilution of sweat components.

### 3.4. Human Sweat Rate and Chloride Ion Detection

**Figure 4:**
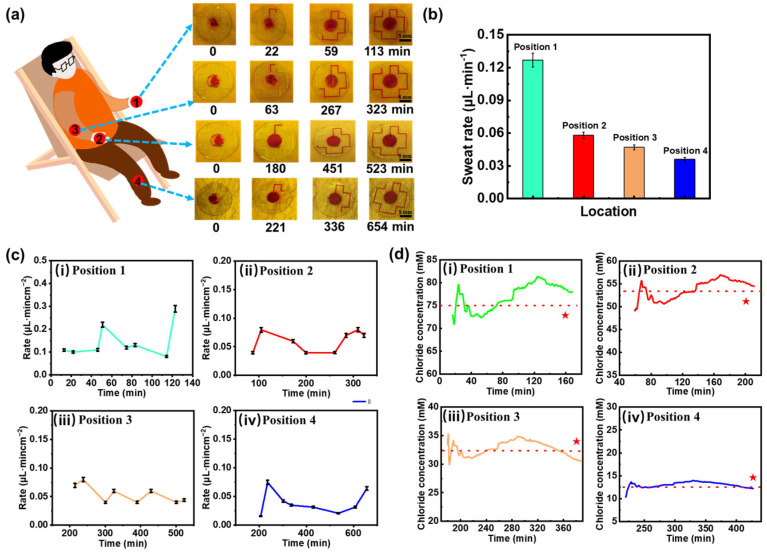
Human sweat rate and chloride ion concentration analysis. (**a**) Microfluidic sweat collection images from various body parts. (**b**) Bar graphs of sweat rate derived from optical imaging data. (**c**) Sweat rate variation curves for different body regions: (i) palm, (ii) back of hand, (iii) arm and (iv) calf. (**d**) Chloride ion concentration variations across different body regions: (i) palm, (ii) back of hand, (iii) arm and (iv) calf. Red stars denote validation results from a commercial Cl⁻ detector, and the red dotted line represents the average measured value across all tested regions.

Sweat-sensing patches embedded with red-dyed hydrogel sheets were applied to the palm (Position 1), back of hand (Position 2), arm (Position 3), and calf (Position 4) of resting subjects for optical image acquisition. Figure 4a displays the optical images of the sweat patches on different body parts, revealing that the palm has the highest natural sweat secretion rate, which can fill the entire channel within two hours. In contrast, the calf has the lowest sweat rate, requiring approximately 11 h for collection. Figure 4b sweat rate histogram obtained from the optical image and Equation (2) shows a peak sweat rate at the palm of the hand of approximately 0.13 μL·min^−1^. The sweat rate histogram derived from the optical image shown in Figure 4b and indicates a peak sweat rate of about 0.13 μL·min^−1^ on the palm. These experimental results illustrate the feasibility of using microfluidic sensing patches based on hydrogel interface for sweat extraction and collection in a stationary state. However, optical methods only allow for a rough analysis of average sweat rates across different body parts. To achieve more accurate measurements, this proposed microfluidic sensing patch is employed to continuously monitor real-time pulse-conductance-based sweat rate. As illustrated in Figure 4c, at the onset of sweating, all four monitored sites exhibit an increasing trend in sweat rate, consistent with previous studies reported [51]. Despite being at rest, slight variations in sweat rate may be attributed to heart rate activity or changes in external temperature. The sweating rates in different parts of the body are consistent with previously reported results [52].

While measuring the sweat rate for the aforementioned subjects, Cl^−^ concentration in sweat was simultaneously detected. The Cl^−^ concentration at each measurement point was obtained by correlating the electrode conductance value reaching the trigger point with the linear fitting curve in Figure 3b(iv). The measurement results are presented in Figure 4d, where the red line indicates the average measured value. As expected, the palm, which exhibits the highest sweat rate, also shows the highest average Cl^−^ concentration, approximately 75 mM. Conversely, the calf, characterized by the lowest sweat rate, has the lowest detected Cl^−^ concentration, fluctuating around 13 mM. This suggests that the concentration of Cl^−^ in specific areas of the human body is influenced by the sweating rate. To validate the accuracy of Cl^-^ concentration detection, we conducted tests using a commercial Cl^−^ detector, as indicated by the red stars in the Figure. The results are 73 mM, 51 mM, 34 mM, and 15 mM, respectively, in specific areas of the human body with an experimental error of less than 10%, thereby confirming the accuracy of the microfluidic sweat-sensing patch in detecting chloride ions. Additionally, sweat sensing patches were attached to adjacent locations on the palm of subjects, and the detected sweat rates demonstrate that the trend in sweat rate changes exhibits high consistency, with the curves almost overlapping during periods of sweat rate measurement (Figure S11), indicating that the sweat-sensing patches possess good stability and repeatability.

### 3.5. Human Sweat Glucose Detection

**Figure 5:**
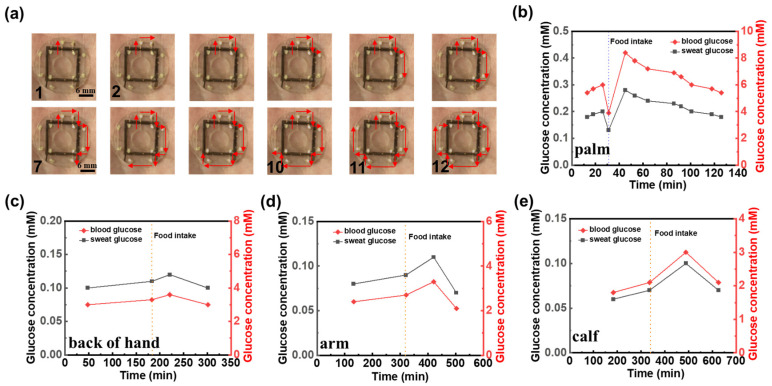
Human sweat glucose detection. (**a**) Optical image of the microfluidic sweat sensing patch for human sweat glucose detection. Comparison of glucose concentrations between commercial blood glucose test and sweat glucose levels detected by microfluidic sweat sensing patch at various human body sites: (**b**) palm, (**c**) back of hand, (**d**) arm, (**e**) calf. Dotted lines in (**b**–**e**) denote the timing of food intake during the measurement period.

Invasive blood sampling for glucose monitoring can induce patient discomfort and anxiety and carries a non-negligible risk of infection. As an alternative, sweat presents a compelling non-invasive medium for metabolic assessment. Although the glucose concentration in sweat is substantially lower than that in blood, previous studies have demonstrated a positive correlation between sweat glucose and blood glucose levels under physiologically stable conditions [53]. Leveraging this relationship, our platform employs a colorimetric assay that enables visual detection of glucose in sweat through quantifiable color changes, offering a convenient, needle-free approach for monitoring glycemic trends. In our system, a colorimetric module was integrated at 12 discrete positions along the microfluidic channel prior to encapsulation of the sensing patch (Figure 1b and Figure 5a). As sweat flows through the channel, it sequentially interacts with each module, inducing a color change at each site. The RGB values of these color changes were then captured using a color picker, allowing us to determine the corresponding glucose concentration by comparing the RGB values with a standard reference curve obtained from the given glucose solution (Figure S12). The palm of the hand, which exhibits the highest sweat rate at rest, was selected for the collection and detection of sweat glucose. As sweat flowed through the color-developing spots on the sensing patch, images were captured using a camera, and the resulting optical images (Figure 5a) were analyzed with a color picker. By referencing the standard curve (Figure S12), the glucose concentration in sweat was determined at each time point (Figure 5b). To explore the correlation between sweat glucose and blood glucose levels, blood glucose was measured simultaneously with the sweat glucose tests using a glucometer (Figure 5b). The results show that the change trends in sweat glucose and blood glucose are similar; however, blood glucose concentrations are approximately 30 times higher than those of sweat glucose. This suggests that sweat glucose levels can serve as an indicator for estimating blood glucose concentrations. Additionally, to investigate the variation of sweat glucose concentrations across different body regions [54], the sweat-sensing patch was applied to the back of the hand, arm, and calf. The experimental results (Figure 5c–e) show that sweat glucose concentrations first increase and then decrease before and after eating, eventually returning to baseline levels, mirroring the trends observed in commercial glucose meter readings. In addition, sweat rate, chloride ion, and glucose were measured in different individuals under the same experimental conditions (Figure S13), and the detection parameters covered normal physiological baseline values. This result indicates that, despite individual baseline differences, sensor detection maintains a stable response over a range of physiological fluctuations.

## 4. Conclusions

In this study, we introduce an attractive hydrogel-based microfluidic platform designed for non-invasive sweat analysis. This platform addresses the critical challenges of inefficient sweat collection and the demand for real-time, multi-biomarker monitoring. By integrating an agarose–glycerol hydrogel sheet within a microfluidic channel, the system facilitates efficient sweat collection without external stimulation. The novel laser-cut double-sided tape structure, embedded with copper electrodes, ensures accurate measurement of sweat rate and chloride ion concentration while minimizing electrochemical interference. Furthermore, the colorimetric glucose detection module enables effective glucose monitoring via a simple and cost-effective RGB analysis. The platform’s capability to analyze a broad spectrum of biomarkers, combined with its wearable design, positions it as a promising solution for personalized healthcare applications, demonstrating its significant potential for continuous, non-invasive health monitoring and opening up new possibilities for diagnostics in both clinical and home settings.

## Data Availability

Data are contained within the article.

## Supplementary Materials

The following supporting information can be downloaded at https://www.mdpi.com/article/10.3390/bios15060342/s1: Figure S1 with sensing patch schematic, microfluidic channel dimensions, and parameter descriptions; Figure S2 shows the comparative experiments of sensor sweat collection with and without hydrogels; Figure S3 showing optical images of hydrogel sheets at 150 μm, 300 μm, and 450 μm thicknesses; Figure S4 shows the mechanical stability and long-term absorption rate stability of the patch; Figure S5 presenting SEM images of channel inner wall roughness under varying laser scanning rates; Figure S6 displaying optical microscopy images of microfluidic channels with different cutting widths; Figure S7 analyzing flow resistance variations influenced by channel height, width, and length; Figure S8 illustrating the measuring system module and partial circuit diagram; Figure S9 depicting electrode admittance under different cation types; Figure S10 outlining the LabVIEW program framework; Figure S11 validating sweat rate measurements using dual adjacent sensing patches; Figure S12 demonstrating colorimetric glucose detection via RGB value changes and ΔR fitting curves under white light illumination; Figure S13 shows the validation of sweat rate, chloride, and glucose detection in different individuals under the same conditions; Movie S1 showcasing the LabVIEW admittance test interface.
