# Supplementary material for: Sweat-Sensing Patches with Integrated Hydrogel Interface for Resting Sweat Collection and Multi-Information Detection
Source: Biosensors (Basel). 2025 May 29;15(6):342. doi: 10.3390/bios15060342 (PMC12190536; doi:10.3390/bios15060342)
Supplement: Supplementary file 1 [file biosensors-15-00342-s001.zip › Supplementary Information.pdf]

Supplementary Information

Sweat Sensing Patches with Integrated Hydrogel Interface for Resting Sweat Collection and Multi-information Detection

Lei Lu[1], Qiang Sun[1], Zihao Lin[1], Wenjie Xu[1], Xiangnan Li[1], Tian Wang[1], Yiming Lu[1], Huaping Wu [2], Lin Cheng [1*] and Aiping Liu [1*]

[1] Zhejiang Key Laboratory of Quantum State Control and Optical Field Manipulation, Department of Physics, Zhejiang Sci-Tech University, Hangzhou 310018, China

[2] Key Laboratory of Special Purpose Equipment and Advanced Processing Technology, Ministry of Education and Zhejiang Province, College of Mechanical Engineering, Zhejiang University of Technology, Hangzhou 310023, China

***Corresponding authors:** chenglin@zstu.edu.cn; liuaiping1979@gmail.com

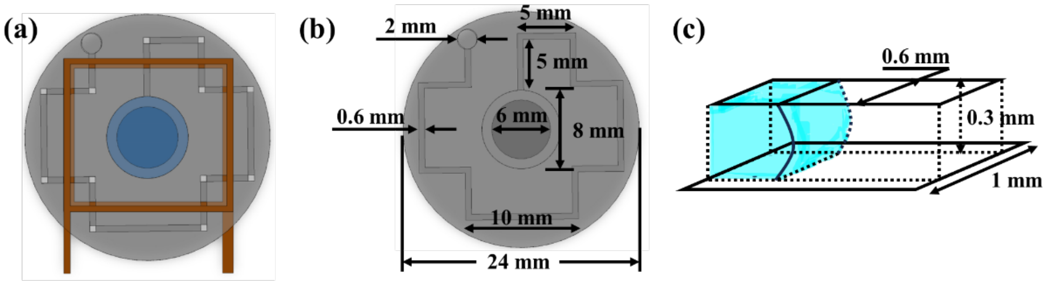

Figure. S1. Dimensions and parameters of sweat sensing patch. (a) Schematic of the sensing patch, (b) dimensions of microfluidic channel, inlet and outlet, (c) parameter description of the rectangular channel.

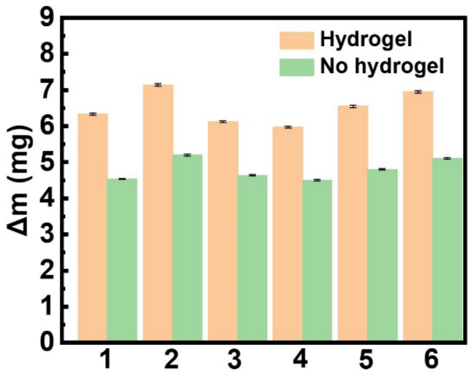

Figure S2. Comparative experiments of sensor sweat collection with and without hydrogels.

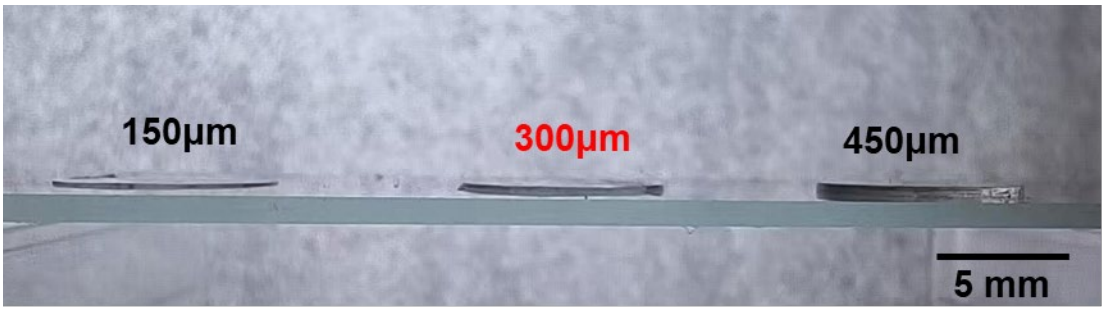

Figure. S3. Optical images of hydrogel sheets with thicknesses of 150 μm, 300 μm and 450 μm, respectively.

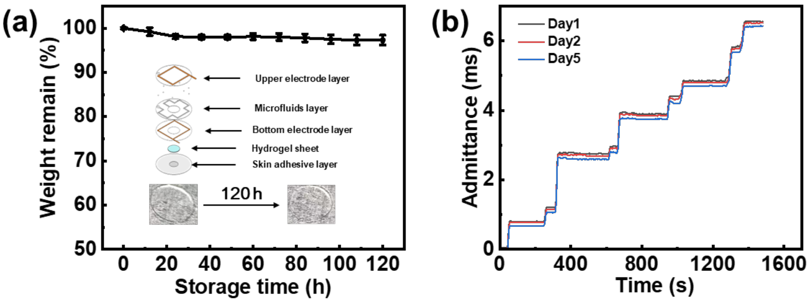

Figure. S4. (a) Weight change of the sensor over 120 hours at room temperature. The inset shows the sensor disassembly diagram and the hydrogel patch optics. (b) Repeatability test of the sensor after different days (1, 3 , 5 days).

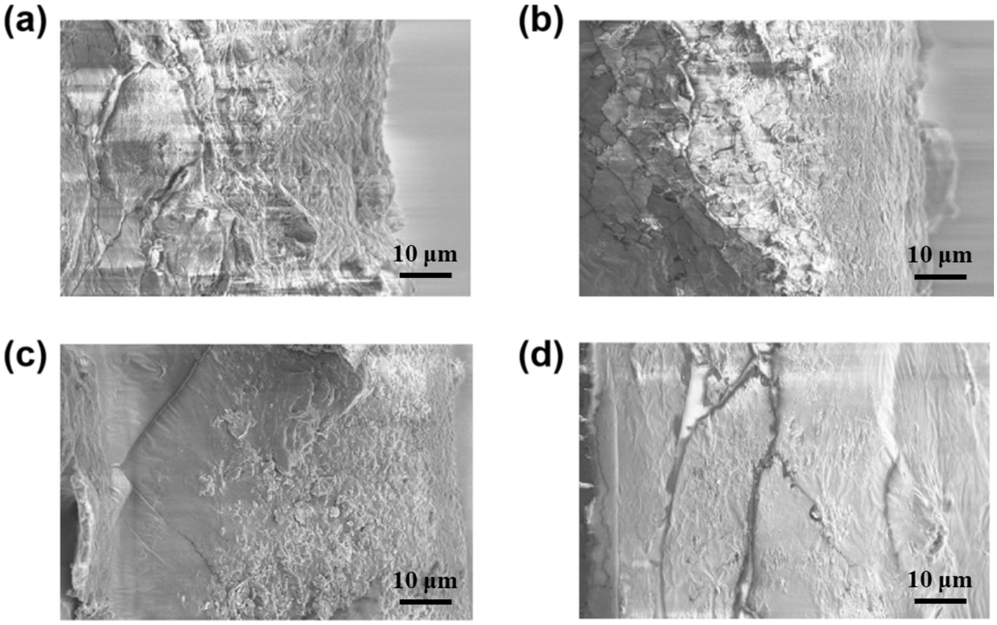

Figure. S5. SEM images illustrating the roughness of microfluidic channel inner walls processed at varying laser scanning rates: (a) 10 mm/s; (b) 30 mm/s; (c) 40 mm/s; (d) 50 mm/s.

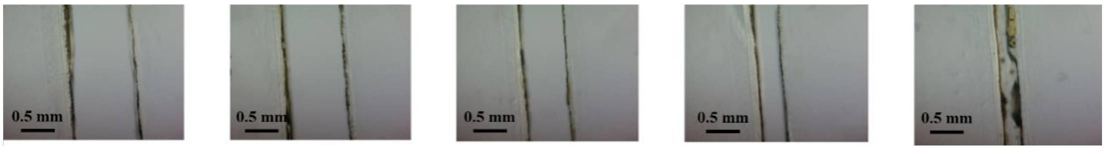

Figure. S6. Optical microscope images of microfluidic channels with widths progressively decreasing from 1 mm, 0.8 mm, 0.6 mm, 0.4 mm to 0.2 mm, from left to right, respectively.

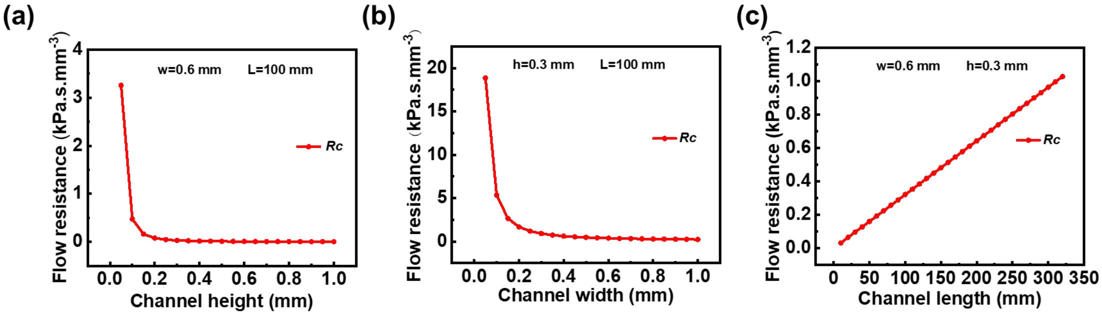

Figure. S7. Variation in flow resistance of microfluidic channels: (a) Effect of w=0.6 mm channel height on flow resistance; (b) the effect of channel width on flow resistance; (c) the influence of channel length on flow resistance.

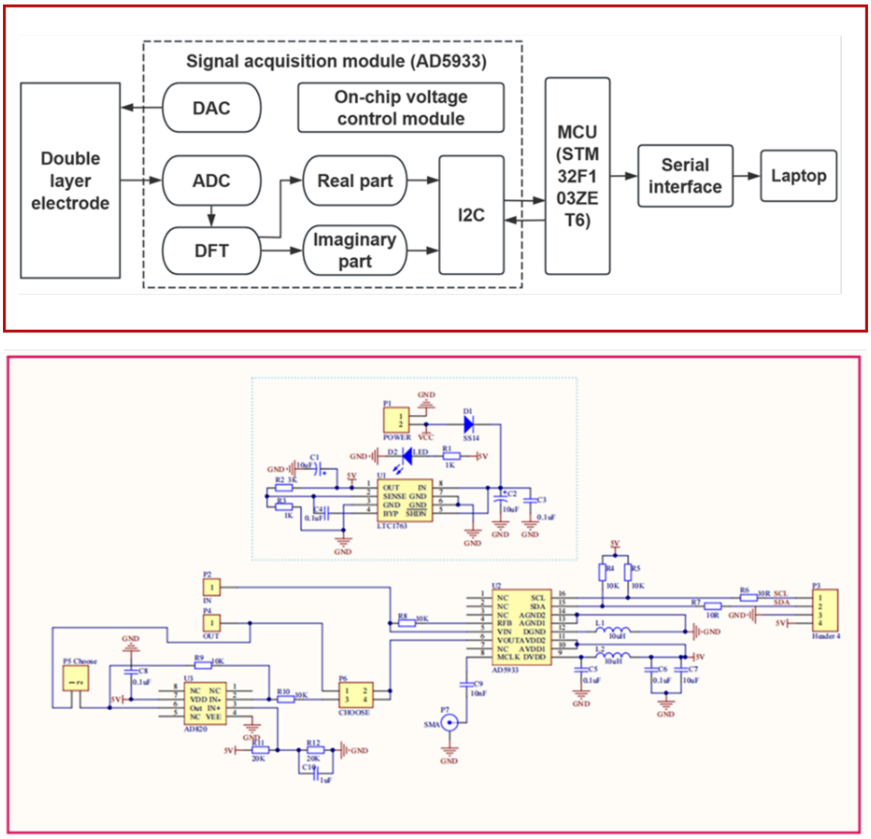

Figure. S8. Schematic diagram of measuring system module and partial circuit diagram.

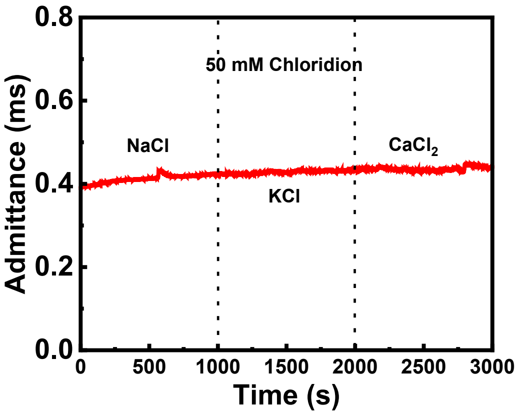

Figure. S9. Electrode admittance under different cation types

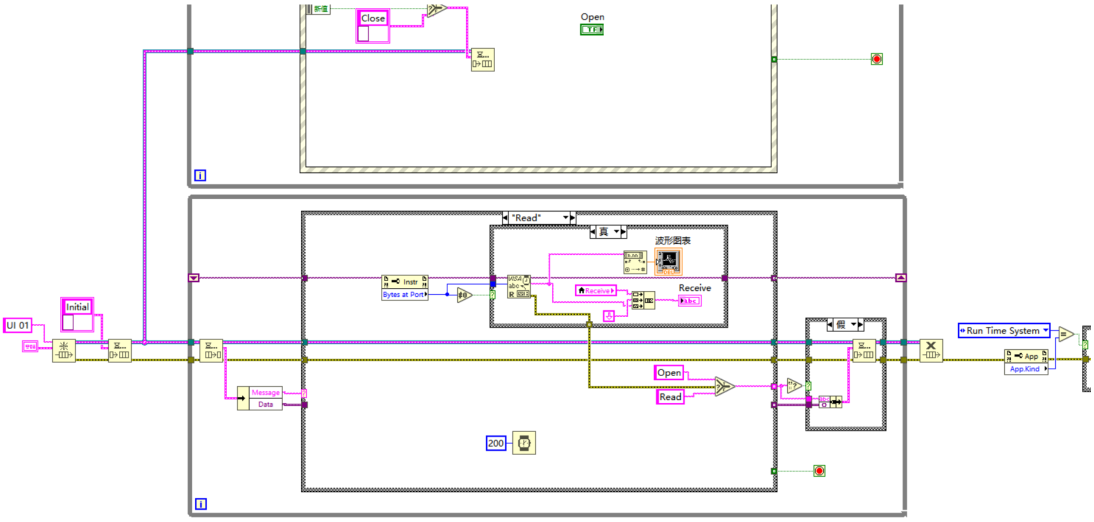

Figure. S10. LabVIEW program framework.

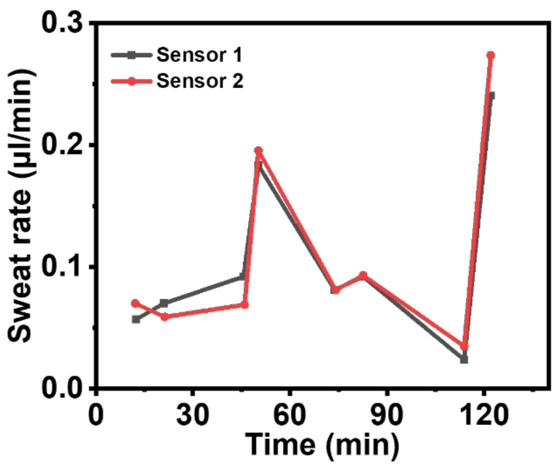

Figure. S11. Sweat rate measured using two adjacent microfluidic sensing patches placed on the palm.

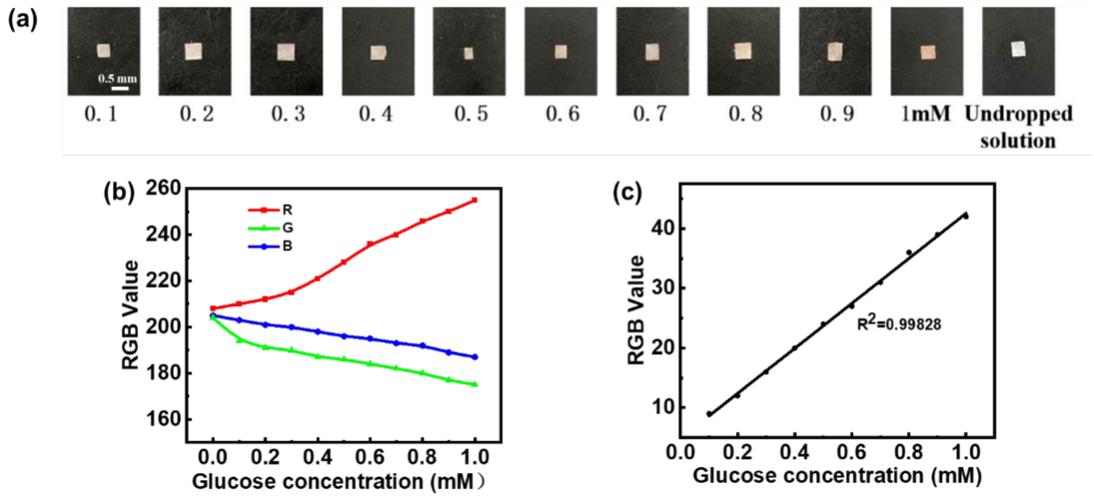

Figure. S12. (a) Optical images depicting the color changes in colorimetric tablets upon the dropwise addition of glucose solutions with given concentrations. (b) RGB value change curve of colorimetric tablets under 2.5W white light illumination. (c) Fitting curve of ΔR for colored square samples under 2.5 W white light illumination.

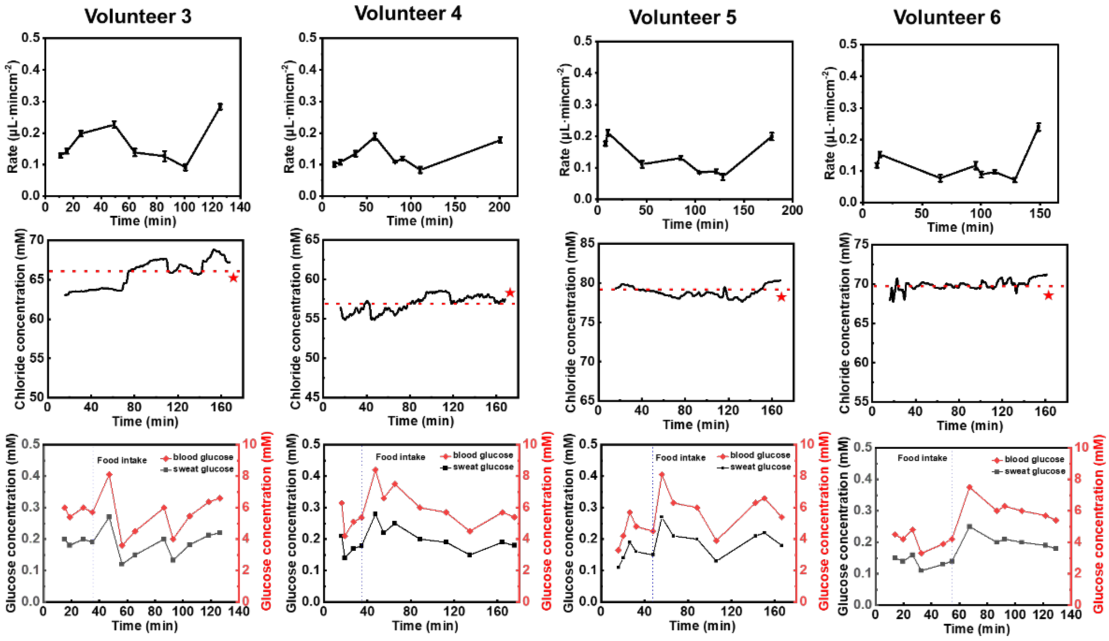

Figure. S13. Verification of sweating rate, chloride ion and glucose detection under the same conditions for different individuals.

Movie S1: LabVIEW display window for the electrode admittance test
